# The Effects of Occupational Stress and Stress Management on the Performance of Frontline Healthcare Service Workers

**DOI:** 10.3390/healthcare14111582

**Published:** 2026-06-04

**Authors:** Ngqabutho Moyo, Anita D. Bhappu

**Affiliations:** Department of Management of Complex Systems, School of Engineering, University of California Merced, 5200 North Lake Rd., Merced, CA 95343, USA; abhappu@ucmerced.edu

**Keywords:** healthcare services, nursing, occupational stress, stress management, employee engagement, turnover intention, workload, role conflict, work complexity, job demands

## Abstract

**Background**: Managing occupational stress in healthcare services is critical because frontline workers operate under multiple job demands. **Objective**: We extend the literature on health psychology and organizational behavior by examining how two types of occupational stress—eustress and psychological distress—impact the performance of frontline healthcare service workers. We also investigate the interactive influence of stress management strategies—savoring and avoidance coping—on the performance effects of occupational stress. **Methods**: We surveyed 400 frontline healthcare service workers across the globe using MTurk. We used Smart PLS4 to assess our measures and test our hypotheses. **Results**: Job demands—a higher-order construct comprising workload, role conflict, and work complexity—had a non-significant effect on eustress (β = 0.037, *p* = 0.596) but a significant positive effect on psychological distress (β = 0.566, *p* < 0.001). Eustress had a positive effect on employee engagement (β = 0.229, *p* < 0.001) and savoring (β = 0.437, *p* < 0.001). Psychological distress had a positive effect on turnover intention (β = 0.275, *p* < 0.001) and avoidance coping (β = 0.525, *p* < 0.001). The interaction between savoring and eustress had a negative effect on employee engagement (β = −0.162, *p* = 0.003). The interaction between avoidance coping and psychological distress had a negative effect on turnover intention (β = −0.058, *p* = 0.054). **Conclusions**: Job demands in frontline healthcare services manifest as hindrance stressors that increase workers’ psychological distress. Avoidance coping is an effective strategy for managing psychological distress and reducing workers’ turnover intention.

## 1. Introduction

Managing the effects of occupational stress on employees is a key priority for organizations given the productivity losses and increased costs associated with this workplace challenge [1]. Stress is simply the human body’s general reaction to any demand [2], whereas occupational stress [3] is specifically experienced by employees and caused or exacerbated by specific aspects of the workplace, work environments, or workplace conditions [3,4,5]. Occupational stress affects employees’ work performance [6,7] and psychological resilience [8]. Since the COVID-19 pandemic, these challenges have intensified [9,10], with global reports indicating that 44% of workers experienced stress in 2021 and 2022 compared to 31% in 2009 and 33% in 2014 [11]. As such, 79% of managers are concerned about employees’ occupational stress because less than 30% of firms have policies and procedures to address it [12].

A lot of attention has been directed toward examining the occupational stress of frontline healthcare workers since the COVID-19 pandemic [13,14], which caused them additional strain and negatively impacted their performance [15]. Close to 50% of healthcare workers reported feeling burned out in 2022, an increase from 32% in 2018 [16]. Occupational stress is particularly salient in healthcare services because frontline employees operate under multiple job demands [17]. Moreover, there is limited understanding of the effectiveness of stress management strategies in this critical decision-making work context [18]. We may therefore be ignoring important mechanisms linking occupational stress to employee performance, as well as the effectiveness of coping strategies in frontline healthcare settings.

In response, this paper extends the literature on health psychology and organizational behavior by examining how the two types of occupational stress—eustress and psychological distress [19]—influence the performance of frontline healthcare service workers. We also investigate the interactive influence of stress management strategies—savoring and avoidance coping [20]—on the performance effects of occupational stress. Accordingly, this study addresses the following research questions: How do eustress and psychological distress influence the performance of frontline healthcare service workers? Are savoring and avoidance coping effective stress management strategies for frontline healthcare service workers?

## 2. Literature Review and Hypotheses

### 2.1. Stress and Stress Management

Stress can be defined as the body’s nonspecific reaction to any demand, whether that demand arises from or leads to positive or negative conditions [21]. Stress is triggered by adverse events that are potentially distressing [22] and can deplete a person’s emotional, physical, and cognitive resources, leading to heightened levels of stress [23]. Prior studies have consistently demonstrated a strong link between various stressors and the experience of stress [24]. Long-term, persistent stressful situations are associated with anxiety and depression [25,26]. Stressful events are prerequisites for stress, and the severity of stress is determined by the context in which it occurs [27].

The Job Demands–Resources model [28] characterizes stress at work as the disruption of employees’ cognitive, emotional, and environmental equilibrium by external factors [28,29]. Increased workloads, organizational changes, and reduced benefits and salaries have all been linked to employee stress [30]. Previous research has also shown that upsetting events and psychosocial pressures at work increase employee stress [24]. These stressful events are recognized as significant drivers of emotional exhaustion and depersonalization among workers [28]. Emotional exhaustion and depersonalization are generally regarded as primary components of employee burnout and are often linked to employee stress [28]. Workers who experience emotional exhaustion often feel overwhelmed, depleted, and drained due to ongoing emotional strain, while depersonalization entails developing a cynical or detached attitude toward work [28]. However, the Job Demands–Resources model [28] also suggests that job demands only negatively impact employees when those demands are high, but their job resources are limited [28]. According to this model, there is a need to balance job demands and resources to mitigate the adverse effects of stressful demands at work [31].

Individuals may either avoid or approach a stressful event depending on the nature of a job demand. When individuals avoid stressors, they are focused on emotional detachment [32,33] and are seeking long-term benefits [33,34]. Avoidance is associated with a person’s motivation to evade unproductive situations and unpleasant consequences [33,35]. Individuals often avoid thinking about traumatic events [33], which enable them to withdraw or retreat from dealing with such situations [33]. Avoidance is therefore useful when individuals’ emotional resources are limited and they cannot effectively cope with additional demands [33]. Research suggests that avoidance is most helpful when the impact of a stressful event is short term [36].

On the other hand, individuals approach stressful events when they have some control over the situation and are seeking short-term benefits [33]. Approach is associated with a person’s motivation to engage with productive situations and rewarding consequences [33,35]. An approach strategy involves exercising vigilance and orientation toward a stressful situation [32,35] and is more effective when the impact of a stressful event is long term [36]. Approach is therefore characterized by the cognitive and emotional acknowledgment of a stressful situation and its consequences [33].

As the detrimental type of occupational stress [21], psychological distress has been the focus of prior research [13,37] investigating the negative associations between workplace demands and employee performance and well-being. Psychological distress is therefore an individual’s negative psychological response to hindrance stressors that are evaluated as unhealthy and dysfunctional [20]. On the other hand, eustress involves an individual’s positive psychological reaction to workplace stressors that are evaluated as healthy and functional [38]. As the functional and beneficial type of occupational stress [20,21], eustress is associated with controllable stressors and situations that require individuals to adapt and push themselves beyond their comfort zones [21,39]. Eustress is therefore an employee’s response to challenge stressors that motivate them [40] and can be situated within the area of positive organizational psychology [19].

Depending on whether employees choose to approach or avoid job demands [35,41], occupational stressors may be evaluated either as challenge stressors or hindrance stressors. Based on the challenge–hindrance stressor framework [42,43], challenge stressors are typically appraised as positive, functional, and motivating work demands that trigger a fight response, resulting in good stress or eustress [20,35]. In contrast, hindrance stressors are appraised as negative, dysfunctional, and depleting work demands [20] that cause a flight response, resulting in bad stress or psychological distress [20,35].

### 2.2. Occupational Stress in Frontline Healthcare Services

Within the frontline healthcare services context, employees’ challenge–hindrance stressor appraisals are consequential because workers operate in high-stress environments characterized by multiple job demands [44,45]. Frontline healthcare service workers provide a variety of clinical services directly to patients and serve as the face of healthcare organizations by defending their image and reputation, as well as dealing with queries, claims, complaints, and bad publicity [46]. Due to unprecedented job demands [44,47], frontline healthcare service workers report high levels of stress [48,49,50,51,52,53], anxiety, depression, burnout, fatigue, strain, and turnover intentions [15,54,55,56,57,58] from their daily interactions with patients. In line with this, we now discuss the common job demands of workload [59], role conflict [60], and work complexity [61] experienced by frontline healthcare workers.

Workload is operationally defined as the amount of work assigned to or expected from an employee within a specified timeframe [62,63]. Numerous studies have shown that increased workload due to multiple factors and decisions can result in adverse outcomes for employees [64], including symptoms such as fatigue [65], depression, and anxiety [66,67]. In these studies, increased workload manifested as a hindrance stressor that increased psychological distress. Frontline healthcare workers do not typically have control over their emergent work environment and, therefore, may not be able to avoid the detrimental occupational stress stemming from patients’ unpredictable and overwhelming needs. In other words, they experience psychological distress because their job stressors are high and their job resources are limited [28]. Frontline healthcare workers consistently report emotional exhaustion—a key element of burnout—due to their workload [57,68,69,70]. Coupled with long working hours, workload has a profound impact on the mental well-being of frontline healthcare workers [71,72]. However, workload may function as a challenge stressor when workers perceive higher task demands as opportunities for productivity, growth, and achievement, which usually occurs when they have sufficient autonomy and job resources [42].

Role conflict [53] can be conceptualized as the compatibility or incompatibility of the requirements of a role, evaluated based on a set of conditions or standards that impact role performance. Workers trying to accomplish conflicting expectations often experience confusion and anxiety [73]. Frontline healthcare workers need clear guidance on the assignment and priority of emergent tasks to minimize the detrimental effects of role conflict on their job performance [74]. Role conflict, therefore, is likely appraised as a hindrance stressor to be avoided [42] and may trigger a flight response as employees attempt to distance themselves from its negative consequences [35]. Role conflict often results in increased fatigue [23], emotional exhaustion, and turnover intentions among frontline healthcare workers [74]. Although role conflict is typically categorized as a hindrance stressor, it can function as a challenge stressor when workers perceive it as manageable, which usually occurs when they have some level of support and autonomy. As a challenge stressor, role conflict is associated with problem solving, role negotiation, and prioritization, thereby enhancing performance [75,76,77,78].

Frontline healthcare workers also experience psychological distress due to the complexity of their work [79]. Work complexity can be understood as a cognitively demanding workplace that imposes a significant mental load on employees and depletes their job resources when increasing task demands exceed available job resources [80,81]. Prior research has shown that frontline service work is often characterized by its complexity and non-linear nature [82]. Frontline healthcare service workers are regularly exposed to physically and emotionally taxing tasks [83,84]. Their roles typically involve cognitive labor, encompassing aspects like on-the-job learning [85], critical thinking [86], clinical reasoning [87], clinical decision-making [88], and organizational responsibilities [82,89]. The psychological distress stemming from the work complexity of providing healthcare services is negatively associated with employee well-being and job performance [42,90]. However, work complexity may function as a challenge stressor when it encompasses cognitively demanding tasks that require problem-solving and learning, which enhances workers’ competence, mastery, and personal development [42,91].

Research suggests that frontline healthcare workers encounter challenge stressors that they approach and hindrance stressors that they avoid [39,42,92,93]. Both types of workplace stressors contribute to these employees’ occupational stress and influence their performance [94], highlighting the importance of understanding their impacts at elevated levels [66,67]. However, the extant literature on occupational stress and workplace stressors lacks research that investigates the potentially beneficial effects of eustress at work [95], although a recent study found a positive correlation between eustress and employee performance [39]. We know that employees experience more occupational stress as their exposure to workplace stressors increases and each additional stressor adds to employees’ stress levels [96]. In other words, there is a cumulative effect of multiple workplace stressors on employees’ occupational stress [96], which is particularly relevant to the high-stress work environment of frontline healthcare services. We therefore posit the following:

**Hypothesis 1:**  *As job demands increase, eustress experienced by frontline healthcare service workers grows.*

**Hypothesis 2:**  *As job demands increase, psychological distress experienced by frontline healthcare service workers grows.*

### 2.3. Occupational Stress and Employee Performance

We now turn our attention to the effects of occupational stress on employee performance [97], which can be understood as the extent to which employees are able to fulfill their job requirements and responsibilities effectively and efficiently [98]. Prior research suggests that psychological distress decreases employees’ ability to perform new tasks, ultimately reducing their pace of work [99]. It makes them feel overwhelmed and powerless [100], which also reduces their ability to perform innovative tasks [39]. As their ability to fully concentrate on the job decreases, employees’ turnover intention may increase [101,102,103]. High levels of psychological distress may also affect employee performance by damaging relationships with their employer and co-workers [7]. Stressed workers tend to view their workplaces as lacking social support and may therefore develop organizational distrust that increases their turnover intention [104,105,106,107]. Psychological distress therefore increases employees’ turnover intention [13,19,108,109]. Turnover intention among employees is concerning because it is associated with actual employee turnover [110,111]. When employees resign, organizations incur significant recruitment and replacement costs that may affect their profitability and performance [110]. Prior research makes a distinction between voluntary and involuntary turnover [112,113]. Voluntary turnover occurs when employees choose to leave their jobs, whereas involuntary turnover occurs when they are forced to leave their jobs due to situations such as dismissals and layoffs [114]. There is no evidence, however, that psychological distress has a differential influence on voluntary and involuntary turnover among employees. We therefore focus on voluntary turnover, which is a performance outcome within employees’ behavioral control.

Eustress, on the other hand, has positive implications for employee performance. Employees experiencing eustress often display increased levels of engagement characterized by increased involvement and self-efficacy [115], as well as enhanced job performance [13,95]. Eustress influences individuals to have a state of mind that is positive and fulfilling, characterized by dedication and commitment [116]. Employees experiencing eustress therefore exhibit concentration and resilience in the execution of their work and develop a sense of organizational attachment and pride despite facing workplace challenges [116]. They are happily engrossed in their work, and time often passes without them even noticing [116]. As such, employees experiencing eustress often display attitudes such as positive affect, meaningfulness, and hope [38,117,118,119,120,121,122], which enhance their performance and engagement at work [123,124,125,126]. They often see opportunities amid challenges [19,127,128,129]. They have a growth mindset, which enhances their work engagement and performance [119,123,125]. They also believe they can overcome challenges with effort, optimism, and perseverance [19,95]. All of these are key characteristics of employee engagement [116]. We therefore posit the following:

**Hypothesis 3:**  *There is a positive effect of eustress on employee engagement such that when eustress increases, employee engagement increases.*

**Hypothesis 4:**  *There is a positive effect of psychological distress on turnover intention such that when psychological distress increases, turnover intention increases.*

When employees experience eustress at work, their ability to enjoy and appreciate it becomes crucial in enhancing their engagement. As eustress occurs, it increases savoring [130]. Savoring is the term used to describe the process of enjoying something rather than enjoyment itself [131]. It is a positive emotional state associated with the appreciation of a challenging situation or experience [95] and, therefore, is the counterpart of coping [131]. Savoring only occurs in the present moment but may focus on the past, present, or future [131]. While savoring eustress, employees demonstrate key characteristics of employee engagement such as increased motivation, enthusiasm, and dedication for their work [116,131,132]. Therefore, prior research has suggested a moderating or amplification effect of savoring on the relationship between eustress and employee engagement [95,131]. We therefore posit the following:

**Hypothesis 5:**  *There is a positive effect of eustress on savoring such that when eustress increases, savoring increases.*

**Hypothesis 6:**  *The positive effect of eustress on employee engagement is moderated by savoring such that when savoring increases, this positive effect increases.*

In contrast, the ability of employees to cope with psychological distress becomes crucial because it increases their turnover intention. Prior research suggests that individuals may respond to psychological distress by engaging or disengaging with it [41]. The origins of their engagement–disengagement argument can be traced back to Cannon’s 1929 formulation of the fight-or-flight response to threat [133]. In this case, the fight response can be regarded as engaging with psychological distress, whereas the flight response can be understood as disengaging from psychological distress [41]. Effective coping strategies (fight response) may lessen the negative impact of psychological distress on employee performance [134], whereas ineffective ones (flight response) prolong psychological distress [41,135,136,137]. We therefore focus on avoidance coping given the considerable criticism leveled against this strategy for failing to effectively mitigate the impacts of psychological distress [134,138]. Avoidance coping is normally used by individuals who are in denial of their stressful situation and negatively affects employees’ performance [109,139]. When resorting to avoidance coping, employees who are experiencing psychological distress often consider quitting their job [19]. In this way, psychological distress increases avoidance coping that amplifies employees’ turnover intention [13]. We therefore posit the following:

**Hypothesis 7:**  *There is a positive effect of psychological distress on avoidance coping such that when psychological distress increases, avoidance coping increases.*

**Hypothesis 8:**  *The positive effect of psychological distress on turnover intention is moderated by avoidance coping such that when avoidance coping increases, turnover intention increases.*

## 3. Materials and Methods

**Figure 1:**
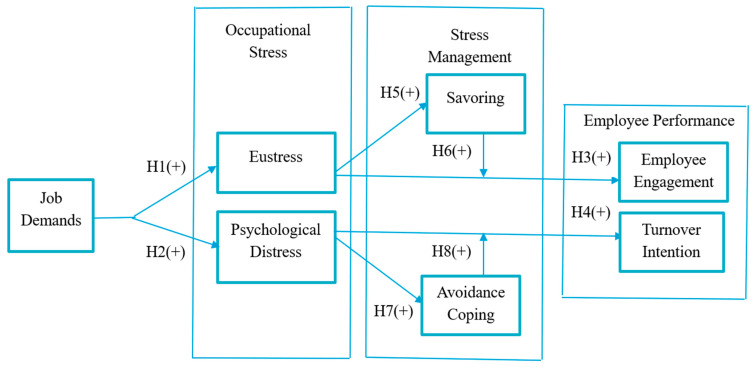
The effects of occupational stress and stress management on the performance of frontline healthcare service workers.

To quantitatively test our theoretical model and hypothesized relationships (see Figure 1), we surveyed frontline healthcare service workers. They were appropriate research participants because they have ongoing exposure to multiple job demands and high levels of occupational stress [140,141,142]. To determine the minimum sample size needed to capture key characteristics of our participant population, we used Slovin’s formula [143,144]. Assuming a population size of 100,000 frontline healthcare service workers globally with a margin of error of 5% (e = 0.05), our required minimum sample size was 399 participants. We therefore recruited a sample of 400 survey participants.

Our survey research protocol was reviewed and approved by the Institutional Review Board (IRB) at a public Canadian university (CU) in the province of British Columbia (BC). All data and materials are publicly available on the Open Science Framework (OSF).

### 3.1. Recruiting Participants

Using purposive, non-probability sampling, we administered an English language cross-sectional survey to 400 frontline healthcare service workers across the globe who we recruited using Amazon Mechanical Turk (MTurk). MTurk is an online crowdsourcing platform for recruiting survey research participants [145]. On the MTurk platform, researchers can recruit participants by posting an online survey as a paid Human Intelligence Task (HIT) [146]. Participants are also afforded anonymity because their survey responses are only linked to their MTurk ID [146].

We used MTurk in our study because it is an efficient, reliable, and cost-effective way to recruit a global sample of frontline healthcare service workers. Using his registered account, the first author posted a HIT advertisement inviting MTurk users to participate in our online survey. The advertisement briefly described our research purpose, as well as the estimated HIT completion time (15 min) and compensation (USD $2.05); this amount was consistent with recommended compensation for similar MTurk tasks [146].

Interested MTurk users were directed via an external link to our study where they first responded to our consent form, then to our screening questionnaire upon providing consent, and finally to our research survey upon being qualified; all of these steps were administered using CU’s SurveyMonkey software. All survey participants were consented MTurk users who self-reported that they were 18 years or older and were employed as a frontline healthcare service worker. They also completed our survey within seven days of opening it. To ensure data quality and participant authenticity, we implemented several screening and verification procedures. We restricted duplicate responses and multiple submissions from the same IP address, used unique completion codes to verify survey submission prior to compensation, and asked screening questions to confirm participants’ age and status as frontline healthcare service workers. We also employed response-time screening to identify and exclude participants who completed the survey too quickly.

### 3.2. Survey Measures

All the scale items of our survey measures are listed in Appendix A.

**Job Demands:** We operationalized job demands as a higher-order construct that was computed as the composite mean score of three distinct lower-order constructs, namely workload, role conflict, and work complexity. *Workload* was measured using a scale developed by [59], which has a reported Cronbach’s alpha of 0.714 and 5-point Likert responses ranging from 1 = strongly agree to 5 = strongly disagree. *Role conflict* was measured using a scale developed by [60], which has a reported Cronbach’s alpha of 0.780 and similar 5-point Likert responses. *Work complexity* was measured using a scale developed by [61], which has a reported Cronbach’s alpha of 0.820 and 5-point Likert responses ranging from 1 = almost never to 5 = almost always.

**Occupational Stress:** *Eustress* was measured using a scale developed by [19], which has a reported Cronbach’s alpha of 0.800 and 5-point Likert responses ranging from 1 = strongly disagree to 5 = strongly agree that were reverse coded. *Psychological distress* was measured using the adapted Psychological Distress scale [147], which has a reported Cronbach’s alpha of 0.930 and 5-point Likert responses ranging from 1 = none of the time to 5 = all of the time.

**Stress Management:** *Savoring* was measured using adapted items from the Savoring Beliefs Inventory [148], which has a reported Cronbach’s alpha of 0.840 for the total scores and 7-point Likert responses ranging from 1 = strongly disagree to 7 = strongly agree. *Avoidance coping* was measured using a scale developed by [149], which has similar 5-point Likert responses and a reported Cronbach’s alpha of 0.810 for psychiatric outpatients and 0.740 for medical students.

**Employee Performance**: *Employee engagement* was measured using the Utrecht Work Engagement scale [150], which has a reported Cronbach’s alpha above 0.720 across different countries and 7-point Likert responses ranging from 0 = never to 6 = always. *Turnover intention* was measured using a scale developed by [151], which has a reported Cronbach’s alpha above 0.750 and 5-point Likert responses ranging from 1 = strongly disagree to 5 = strongly agree.

**Gender**: We also measured the *gender* of our survey participants using 3 categorical responses: male (coded as 1), female (coded as 2), and prefer not to disclose (coded as 3).

### 3.3. Data Analysis

We calculated descriptive statistics using the Statistical Package for Social Sciences (SPSS version 20) software [152]. Using SmartPLS 4 software [153], we then used two-stage Partial Least Squares Structural Equation Modeling (PLS-SEM) to estimate both our measurement and structural models. PLS-SEM is recommended for analyzing complex predictive models that involve latent variables and higher-order constructs with moderation and mediation effects.

## 4. Results

### 4.1. Reliability and Validity of Survey Measures

We assessed the internal consistency, convergent validity, and discriminant validity of our survey measures using Cronbach’s alpha [152,154], composite reliability (Rho_a, and Rho_c), average variance extracted (AVE), and the Heterotrait–Monotrait (HTMT) ratios [155]. We then tested our hypotheses using a bootstrapping procedure with gender as control variable, which produced path coefficients, t-statistics, and *p*-values to evaluate their statistical significance. We also evaluated multicollinearity through Variance Inflation Factor (VIF) values.

### 4.2. First Model Assessment: Lower-Order Constructs of Job Demands

We first assessed a PLS-SEM model comprising lower-order constructs of workload, role conflict, and work complexity—all job demands. Our results (see Table 1) indicate that these measures had Cronbach’s alphas above 0.600 which were close to previously reported values, indicating acceptable levels of internal consistency [152,156]. Similarly, their Rho_a and Rho_c values exceeded the recommended threshold of 0.700, confirming satisfactory composite reliability. However, the average variance extracted (AVE) for role conflict, eustress, employee engagement, and avoidance coping were below the threshold of 0.500 [155], indicating weak convergent validity. In addition, HTMT values between role conflict, workload, and work complexity were excessively high, indicating poor discriminant validity. Accordingly, we iteratively deleted items with low factor loadings (RC 1 and 5; EU 1, 3 and 4; PD 8; WL 1; AVO 1, 2, 3 and 7) to improve construct validity and overall model quality [157]. Deleting these items increased AVE values to above the 0.500 threshold, except for employee engagement and eustress, which had AVE values slightly below 0.500 but were still acceptable because their composite reliability was above 0.600 [158,159]. However, the HTMT values between role conflict, workload, and work complexity remained excessively high (see Table 2), suggesting a lack of discriminant validity among these measures [155].

### 4.3. Second Model Assessment: Higher-Order Construct of Job Demands

We then assessed a PLS-SEM model comprising a higher-order construct of job demands based on the lower-order constructs of workload, role conflict, and work complexity. This second model (see Table 3) demonstrated acceptable internal consistency and composite reliability with Cronbach’s alpha values exceeding 0.600 [160] and Rho_a and Rho_c values exceeding 0.700 for all measures. AVE values for all measures were also near or above 0.500 and supported by composite reliability values above 0.600 [158,159]. HTMT values were also below 0.900 (see Table 4), indicating adequate discriminant validity for all measures [155].

Calculated values for the Fornell Lacker Criterion (see Table 5) also indicate generally acceptable discriminant validity across the model [159]. Additionally, calculated values for the Variance Inflation Factor (VIF) ranged from 1.22 to 2.33, indicating that multicollinearity is not a concern because these values were well below the acceptable threshold of 5.00 [153].

### 4.4. Descriptive Statistics

We calculated sample means, standard deviations, and bi-variate Pearson correlation coefficients for all our survey measures (see Table 6). Although significant correlations indicate conceptual proximity among some measures, these values remain within the acceptable range of −1 to +1 [161], with no indication of multicollinearity concerns. However, our control variable of gender was significantly correlated with some measures. In our survey sample of 400 participants, 270 (67.5%) were male, 128 (32.0%) were female, and 2 (0.5%) preferred not to disclose. The significant negative correlation between job demands and gender indicates that male participants reported higher job demands than female participants (M_m_ = 3.662; M_f_ = 3.412). The significant negative correlation between psychological distress and gender indicates that male participants reported higher psychological distress than female participants (M_m_ = 2.834; M_f_ = 2.561). The significant negative correlation between avoidance coping and gender indicates that male participants reported higher avoidance coping than female participants (M_m_ = 3.670; M_f_ = 3.502). The significant correlation between gender and turnover intention indicates that male participants reported higher turnover intention than female participants (M_m_ = 3.464; M_f_ = 2.878).

### 4.5. Hypothesis Testing

To test our hypotheses, we used a SmartPLS-SEM bootstrapping technique to assess the significance of the various path coefficients in our measurement model (see Table 7). Hypothesis 1 predicted a positive effect of job demands on eustress. The results indicate that Hypothesis 1 was unsupported because the effect of job demands on eustress was not statistically significant. Hypothesis 2 predicted a positive effect of job demands on psychological distress. The results indicate that Hypothesis 2 was supported because the effect of job demands on psychological distress was positive and statistically significant. Hypothesis 3 predicted a positive effect of eustress on employee engagement. The results indicate that Hypothesis 3 was supported because the effect of eustress on employee engagement was positive and statistically significant. Hypothesis 4 predicted a positive effect of psychological distress on turnover intention. The results indicate that Hypothesis 4 was supported because the effect of psychological distress on turnover intention was positive and statistically significant. Hypothesis 5 predicted a positive effect of eustress on savoring. The results indicate that Hypothesis 5 was supported because the effect of eustress on savoring was positive and statistically significant. Hypothesis 6 predicted a positive moderating effect of savoring on the relationship between eustress and employee engagement. The results indicate that Hypothesis 6 was only partially supported because although the moderating effect of savoring on the relationship between eustress and employee engagement was statistically significant, it was negative. Hypothesis 7 predicted a positive effect of psychological distress on avoidance coping. The results indicate that Hypothesis 7 was supported because the effect of psychological distress on avoidance coping was positive and statistically significant. Hypothesis 8 predicted a positive moderating effect of avoidance coping on the relationship between psychological distress and turnover intention. The results indicate that Hypothesis 8 was only partially supported because although the moderating effect of avoidance coping on the relationship between psychological distress and turnover intention was statistically significant, it was negative.

### 4.6. Gender Effects

**Figure A1:**
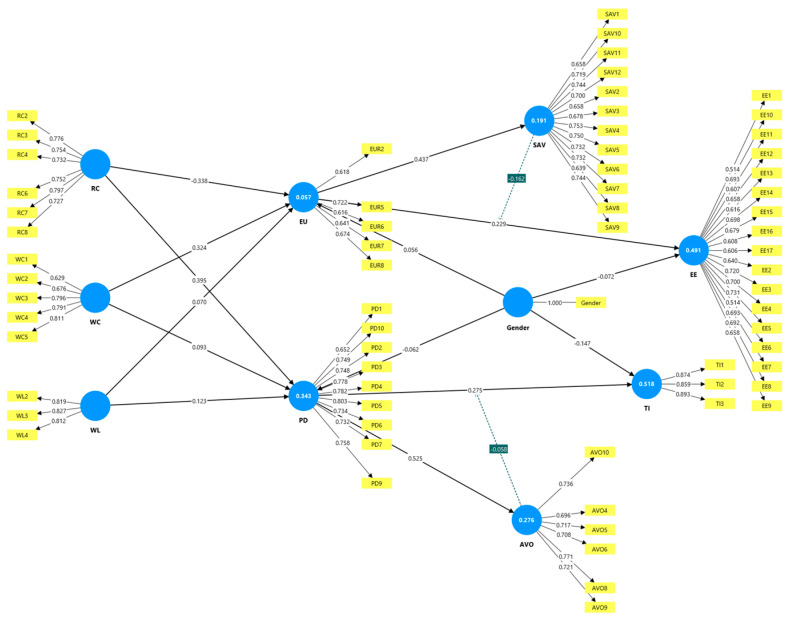
First model assessment: the effects of lower-order constructs.

**Figure A2:**
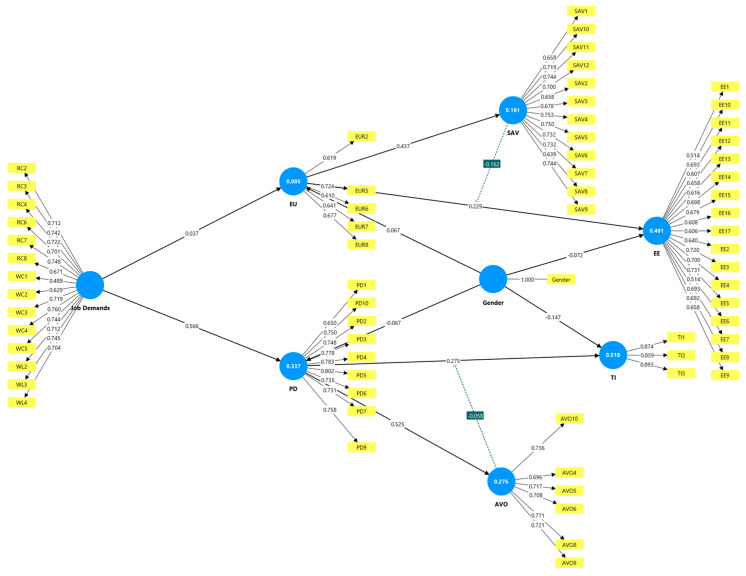
Second model assessment: effect of higher-order construct.

Gender was included as a control variable with direct structural paths mapped to eustress, psychological distress, employee engagement, and turnover intention (see Figure A1 and Figure A2). Gender had limited explanatory power as a covariate (see Table 8) and did not meaningfully alter the structural model. It had no effect on employee engagement, eustress, and psychological distress. It only had a significant negative effect on turnover intention.

### 4.7. Simple Slope Analyses

The interaction between savoring and eustress on employee engagement was negative and statistically significant at both low and high levels of savoring (see Table 9), which indicates that savoring attenuated the positive effect of eustress on employee engagement. The interaction between avoidance coping and psychological distress was negative and statistically significant at both low and high levels of avoidance coping, which indicates that avoidance coping attenuated the positive effect of psychological distress on turnover intention.

## 5. Discussion of Results

Taken as a whole, our survey results highlight how the multiple job demands of frontline healthcare service workers influence their occupational stress and performance, as well as the effectiveness of stress management strategies in this critical decision-making work context.

### 5.1. Job Demands

Hypothesis 1 was unsupported, which suggests that the job demands of workload, role conflict, and work complexity in frontline healthcare services do not manifest as challenge stressors. Hypothesis 2 was supported, however, which suggests that these same job demands in frontline healthcare services instead manifest as hindrance stressors. These two results align with prior research [13,66,67] which found that frontline healthcare workers consistently experience high levels of anxiety, depression, and burnout, which contributes to their psychological distress. They also reinforce recent findings about the pharmacy workforce [162], which indicate that burnout, stress, and workload are major determinants of these frontline healthcare service workers’ satisfaction and retention.

### 5.2. Eustress and Savoring

Hypothesis 3 was supported, which suggests that frontline healthcare service workers who experience higher levels of eustress (possibly because they see opportunities amid challenges [19]) have higher employee engagement. This result aligns with prior research [20,95] which found that eustress—good occupational stress—is associated with positive performance outcomes. Hypothesis 5 was supported, which suggests that frontline healthcare service workers engage more in savoring as their eustress increases. This result aligns with prior research [130] which found that eustress was associated with savoring. Hypothesis 6 was only partially supported, which suggests that savoring attenuated rather than augmented the significant positive effect of eustress on employee engagement. This result contradicts prior research [131] which found that savoring has an amplification effect on the relationship between eustress and employee engagement. It suggests that savoring may increase the cognitive load of frontline healthcare service workers when managing their occupational stress, thereby decreasing their employee engagement.

### 5.3. Psychological Distress and Avoidance Coping

Hypothesis 4 was supported, which suggests that frontline healthcare service workers who experience higher levels of psychological distress have higher turnover intention. This result aligns with prior research [20,103] which found that psychological distress—bad occupational stress—is associated with negative performance outcomes. Hypothesis 7 was supported, which suggests that frontline healthcare service workers engage more in avoidance coping as their psychological distress increases. This result aligns with prior research [28,41] which found that workers use avoidance coping to deal with occupational stress, especially when job resources are limited. Hypothesis 8 was only partially supported, which suggests that avoidance coping attenuated rather than augmented the significant positive effect of psychological distress on turnover intention. This result contradicts prior research [163,164] which found that avoidance coping has an amplification effect or no effect on the negative performance effects of psychological distress. It suggests that avoidance coping may decrease the cognitive load of frontline healthcare service workers when managing their occupational stress, thereby decreasing their turnover intention.

## 6. Limitations and Future Directions

We relied on self-reported survey measures, which may be prone to common method bias [165]. Data collected via MTurk may also be prone to response bias [166] because participants on this platform can provide inaccurate responses [166]. Given that frontline healthcare service workers are predominantly female [167], the high percentage of males in our survey sample may have introduced systematic bias and limits the generalizability of our findings. Although survey participants self-reported being 18 years of age or older, we did not measure their age, job title, years of work experience, or country of origin. Future research should measure these demographics and assess their influence on occupational stress and its associated outcomes. Moreover, participants’ cross-sectional survey responses may have been influenced by their current work perceptions plus personal and emotional states [168], thereby increasing the risk of common method and affective state bias [169,170]. To achieve more reliable and stronger causal inferences, future research should rely on longitudinal survey data from recruited participants with verifiable jobs in healthcare organizations, as well as in other sectors to assess whether our findings generalize to different occupational environments. Future research should also examine the underlying mechanisms by which savoring and avoidance coping attenuate the effects of occupational stress on employee performance.

## 7. Contributions and Conclusions

In this research study, we extended the literature on health psychology and organizational behavior to frontline healthcare services by examining how the two types of occupational stress—eustress and psychological distress—impact worker performance. We also investigated the interactive influence of stress management strategies—savoring and avoidance coping—on the performance effects of occupational stress in this work context. In doing so, we shed light on how occupational stress is associated with employee performance in frontline healthcare services, as well as the effectiveness of stress management strategies for tackling this workplace challenge.

We find that multiple job demands—workload, role conflict, and work complexity—in frontline healthcare services manifest as hindrance stressors that increase workers’ psychological distress. These same job demands do not, however, manifest as challenge stressors that increase workers’ eustress. Avoidance coping decreases the positive effect of psychological distress on their turnover intention, making it an effective strategy for managing this bad occupational stress. However, savoring decreases the positive effect of eustress on their employee engagement, making it an ineffective strategy for managing this good occupational stress.

## Figures and Tables

**Table 1:** Reliability and validity from first model assessment.

Measure	Cronbach’s Alpha	Rho_a	Rho_c	AVE
Role Conflict (RC)	0.851	0.858	0.889	0.573
Work Complexity (WC)	0.797	0.817	0.860	0.554
Workload (WL)	0.756	0.757	0.860	0.672
Eustress (EU)	0.667	0.667	0.789	0.429
Savoring (SAV)	0.910	0.912	0.924	0.504
Psychological Distress (PD)	0.902	0.903	0.920	0.562
Avoidance Coping (AVO)	0.819	0.821	0.869	0.526
Employee Engagement (EE)	0.914	0.920	0.926	0.425
Turnover Intention (TI)	0.848	0.854	0.908	0.766

**Table 2:** Discriminant validity (HTMT) from first model assessment.

Measure	RC	WC	WL	EU	SAV	PD	AVO	EE
RC								
WC	0.915							
WL	0.944	0.943						
EU	0.148	0.214	0.130					
SAV	0.337	0.478	0.383	0.558				
PD	0.641	0.571	0.602	0.168	0.236			
AVO	0.896	0.846	0.847	0.131	0.395	0.609		
EE	0.346	0.444	0.340	0.587	0.683	0.213	0.339	
TI	0.851	0.822	0.834	0.097	0.269	0.620	0.790	0.218

RC = role conflict; WC = work complexity; WL = workload; EU = eustress; SAV = savoring; PD = psychological distress; AVO = avoidance coping; EE = employee engagement; TI = turnover intention.

**Table 3:** Reliability and validity from second model assessment.

Measure	Cronbach’s Alpha	Rho_a	Rho_c	AVE
JD	0.920	0.926	0.931	0.494
EU	0.667	0.668	0.789	0.430
SAV	0.910	0.912	0.924	0.504
PD	0.902	0.904	0.920	0.562
AVO	0.819	0.821	0.869	0.526
EE	0.914	0.920	0.926	0.425
TI	0.848	0.853	0.908	0.766

JD = job demands; EU = eustress; SAV = savoring; PD = psychological distress; AVO = avoidance coping; EE = employee engagement; TI = turnover intention.

**Table 4:** Discriminant validity (HTMT) from second model assessment.

Measure	JD	EU	SAV	PD	AVO	EE
JD						
EU	0.171					
SAV	0.406	0.558				
PD	0.623	0.168	0.236			
AVO	0.890	0.131	0.395	0.609		
EE	0.388	0.587	0.683	0.213	0.339	
TI	0.858	0.097	0.269	0.620	0.790	0.218

JD = job demands; EU = eustress; SAV = savoring; PD = psychological distress; AVO = avoidance coping; EE = employee engagement; TI = turnover intention.

**Table 5:** Fornell Lacker Criterion from second model assessment.

Measure	JD	EU	SAV	PD	AVO	EE	IT
JD	0.703						
EU	0.025	0.655					
SAV	0.354	0.437	0.71				
PD	0.577	−0.103	0.203	0.749			
AVO	0.774	0.069	0.338	0.525	0.725		
EE	0.323	0.476	0.634	0.158	0.279	0.652	
TI	0.764	−0.052	0.231	0.546	0.662	0.134	0.875

JD = job demands; EU = eustress; SAV = savoring; PD = psychological distress; AVO = avoidance coping; EE = employee engagement; TI = turnover intention.

**Table 6:** Descriptive statistics (N = 400).

Measure	Mean	SD	Gender	JD	EU	SAV	PD	AVO	EE
Gender	1.330	0.481	1						
JD	3.582	0.739	−0.152 **	1					
EU	4.068	0.488	0.049	0.033	1				
SAV	5.347	0.958	0.023	0.389 **	0.427 **	1			
PD	2.745	0.794	−0.160 **	0.559 **	−0.084	0.219 **	1		
AVO	3.614	0.678	−0.123 *	0.818 **	0.121 *	0.471 **	0.521 **	1	
EE	5.123	0.917	−0.021	0.357 **	0.447 **	0.623 **	0.185 **	0.370 **	1
TI	3.278	1.093	−0.234 **	0.753 **	−0.046	0.235 **	0.520 **	0.663 **	0.154 **

Significant at the ** 0.01 level (2-tailed) or * 0.05 level (2-tailed). JD = job demands; EU = eustress; SAV = savoring; PD = psychological distress; AVO = avoidance coping; EE = employee engagement; TI = turnover intention.

**Table 7:** Hypothesis testing.

Model Path	Path Coefficient (β)	T Statistics	*p* Values	2.50%	97.50%
JD → EU (H1)	0.037	0.530	0.596	−0.077	0.208
JD → PD (H2)	0.566	14.032	0.000 **	0.483	0.644
EU → EE (H3)	0.229	4.181	0.000 **	0.105	0.335
PD → IT (H4)	0.275	5.918	0.000 **	0.162	0.353
EU → SAV (H5)	0.437	6.961	0.000 **	0.321	0.562
SAV × EU → EE (H6)	−0.162	2.932	0.003 **	−0.230	−0.018
PD → AVO (H7)	0.525	12.457	0.000 **	0.459	0.626
AVO × PD → TI (H8)	−0.058	1.931	0.054 *	−0.120	−0.012

Significant at the ** 0.01 level (2-tailed) or * 0.05 level (2-tailed). JD = job demands; EU = eustress; SAV = savoring; PD = psychological distress; AVO = avoidance coping; EE = employee engagement; TI = turnover intention.

**Table 8:** Gender effects.

Model Path	Path Coefficient (β)	T Statistics	*p* Values	2.50%	97.50%
Gender → EE	−0.072	1.911	0.056	−0.139	0.009
Gender → EU	0.067	1.268	0.205	−0.038	0.173
Gender → TI	−0.147	3.907	0.000 **	−0.206	−0.057
Gender → PD	−0.067	1.916	0.055	−0.141	−0.004

Significant at the ** 0.01 level (2-tailed). EE = employee engagement; EU = eustress; TI = turnover intention; PD = psychological distress.

**Table 9:** Simple slope analyses.

Model Path	Path Coefficient (β)	T Statistics	*p* Values	95.0% (CI) Lower Bound	95.0% (CI) Upper Bound
Low SAV × EU = EE	−0.187	−3.871	0.000	−0.359	−0.117
High SAV × EU = EE	−0.190	−3.871	0.000	−0.359	−0.117
Low AVO × PD = TI	−0.131	−2.248	0.025	−0.285	−0.019
High AVO × PD = TI	−0.098	−2.248	0.025	−0.285	−0.019

SAV = savoring; EU = eustress; EE = employee engagement; AVO = avoidance coping; PD = psychological distress; TI = turnover intention.

## Data Availability

All data and materials are publicly available on the Open Science Framework (OSF) at https://osf.io/r4bnd/overview?view_only=baddda90bd64440894de8d750accfcb5 (accessed on 28 April 2026).

## Abbreviations

The following abbreviations are used in this manuscript:

JD	Job Demands
EU	Eustress
PD	Psychological Distress
SAV	Savoring
AVO	Avoidance Coping
EE	Employee Engagement
TI	Turnover Intention
WL	Workload
RC	Role Conflict
WC	Work Complexity

## Appendix A

**Table A1:** Survey measures.

Construct	Item Code	Item
Job Demands	WL1	I never seem to have enough time to get everything done at work
	WL2	The amount of work I am expected to do is too much
	WL3	My daily activities are affected because of workload
	WL4	I have no energy left at the end of the working day
	RC1	I have to do things that should be done differently
	RC2	I have to work on unnecessary things
	RC3	I receive an assignment without the proper manpower to complete it
	RC4	I receive an assignment without adequate resources and materials to execute it
	RC5	I work with two or more groups who operate quite differently
	RC6	I have to break a rule or policy in order to carry out an assignment
	RC7	I receive incompatible requests from two or more people
	RC8	I do things that are apt to be accepted by one person and not accepted by others
	WC1	I work with many different work tasks at the same time
	WC2	The pre-requisites for my work change which have consequences for what to do next
	WC3	I work under high time pressure due to a heavy workload
	WC4	There is no time to work continuously with more comprehensive tasks
	WC5	There are disturbing interruptions in my work
Eustress	EUR1	I feel excited
	EUR2	I feel determined
	EUR3	I sense I am energetic
	EUR4	I sense I am physically in shape
	EUR5	I sense I am full of life
	EUR6	I am ready to act!
	EUR7	I am willing to expand efforts on my work
	EUR8	I am prepared to engage in my work
Psychological Distress	PD1	Did you feel tired out for no good reason?
	PD2	Did you feel nervous?
	PD3	Did you feel so nervous that nothing could calm you down?
	PD4	Did you feel hopeless?
	PD5	Did you feel restless or fidgety?
	PD6	Did you feel so restless you could not sit still?
	PD7	Did you feel depressed?
	PD8	Did you feel that everything is an effort?
	PD9	Did you feel so sad that nothing could cheer you up?
	PD10	Did you feel worthless?
Savoring	SAV1	Get pleasure from looking forward
	SAV2	Can feel the joy of anticipation
	SAV3	Can enjoy events before they occur
	SAV4	Can feel good by imagining outcome
	SAV5	Know how to make the most of good time
	SAV6	Can prolong enjoyment by own effort
	SAV7	Enjoy looking back on happy times
	SAV8	Feel fully able to appreciate good things
	SAV9	Find it easy to enjoy self when want to
	SAV10	Like to store memories for later recall
	SAV11	Can feel good by remembering past
	SAV12	Easy to rekindle joy from happy memories
Avoidance Coping	AVO1	Went on as if nothing had happened
	AVO2	Felt bad that I couldn’t avoid the problem
	AVO3	Kept my feelings to myself
	AVO4	Slept more than usual
	AVO5	Got mad at the people or things that caused the problem
	AVO6	Tried to forget the whole thing
	AVO7	Tried to make myself feel better by eating, drinking, smoking, taking medications
	AVO8	Avoided being with people in general
	AVO9	Kept others from knowing how bad things were
	AVO10	Refused to believe it had happened
Employee Engagement	EE1	At work, I feel bursting with energy
	EE2	At my job, I feel strong and vigorous
	EE3	When I get up in the morning, I feel like going to work
	EE4	I can continue working for very long periods at a time
	EE5	At my job, I am very resilient, mentally
	EE6	At my work I always persevere, even when things do not go well
	EE7	I find the work that I do full of meaning and purpose
	EE8	I am enthusiastic about my job
	EE9	My job inspires me
	EE10	I am proud on the work that I do
	EE11	To me, my job is challenging
	EE12	Time flies when I’m working
	EE13	When I am working, I forget everything else around me
	EE14	I feel happy when I am working intensely
	EE15	I am immersed in my work
	EE16	I get carried away when I’m working
	EE17	It is difficult to detach myself from my job
Turnover Intention	TI1	I often think of leaving the organization
	TI2	I intend to look for a new job within the next year
	TI3	If I could choose again, I would not work for this organization
